# Biased eviction of variant histone H3 nucleosomes triggers biofilm growth in *Candida albicans*

**DOI:** 10.1128/mbio.02063-23

**Published:** 2023-09-28

**Authors:** Priya Brahma, Rashi Aggarwal, Kaustuv Sanyal

**Affiliations:** 1 Molecular Mycology Laboratory, Molecular Biology and Genetics Unit, Jawaharlal Nehru Centre for Advanced Scientific Research, Bangalore, Karnataka, India; The University of Texas Health Science Center at Houston, Houston, Texas, USA

**Keywords:** *Candida albicans*, variant histone H3, H3V^CTG^, nucleosome eviction, biofilm

## Abstract

*Candida albicans* is an opportunistic human pathogen that colonizes the gastrointestinal and genitourinary tracts of healthy individuals. *C. albicans* yeast cells can switch to filamentous forms. On biotic and abiotic surfaces, the planktonic free-floating yeast cells often form biofilms, a multi-drug-resistant three-dimensional community of yeast and filamentous cells. While alterations in gene expression patterns during planktonic to biofilm growth transitions in *C. albicans* have been studied, the underlying molecular mechanisms largely remain unexplored. Previously, we identified a histone H3 variant (H3V^CTG^), which acts as a negative regulator of biofilm growth in *C. albicans*. In the current study, we performed genome-wide profiling of H3V^CTG^ nucleosomes in *C. albicans* planktonic cells and found them to be enriched at promoter regions. In planktonic cells, H3V^CTG^-enriched regions are mostly devoid of histone H3 post-translational modifications that allow active transcription, thus strengthening the role of H3V^CTG^ as a negative regulator of biofilm formation. By combining genome-wide transcriptional alterations, nucleosome positioning (MNase-seq), and DNA accessibility (ATAC-seq) assays, we show a significant reduction in the total number of nucleosomes in biofilm cells as compared to planktonic cells indicating a more open chromatin state during biofilm growth. Finally, we propose that H3V^CTG^-nucleosome eviction at promoters of biofilm-relevant genes in biofilm-grown cells contributes to achieve the open chromatin state by facilitating easy promoter access of master regulators (activators and repressors) for modulation of gene expression observed during growth phase transitions.

**IMPORTANCE:**

*Candida albicans* lives as a commensal in most healthy humans but can cause superficial skin infections to life-threatening systemic infections. *C. albicans* also forms biofilms on biotic and abiotic surfaces. Biofilm cells are difficult to treat and highly resistant to antifungals. A specific set of genes is differentially regulated in biofilm cells as compared to free-floating planktonic cells of *C. albicans*. In this study, we addressed how a variant histone H3V^CTG^, a previously identified negative regulator of biofilm formation, modulates gene expression changes. By providing compelling evidence, we show that biased eviction of H3V^CTG^ nucleosomes at the promoters of biofilm-relevant genes facilitates the accessibility of both transcription activators and repressors to modulate gene expression. Our study is a comprehensive investigation of genome-wide nucleosome occupancy in both planktonic and biofilm states, which reveals transition to an open chromatin landscape during biofilm mode of growth in *C. albicans*, a medically relevant pathogen.

## INTRODUCTION

*Candida albicans* is one of the most prominent fungal species in the gut microbiome of healthy individuals and lives as a commensal on the mucosal membranes of the human reproductive system, gastrointestinal tract, urinary tract, mouth, skin, and other organs (1, 2). *C. albicans* turns into an opportunistic pathogen that overcomes the host immune system, causing life-threatening infections in immunosuppressed patients. The clinically relevant morphotypes of *C. albicans* present inside the host are yeast, hyphae, and pseudohyphae (2 – 4). Phenotypic switching of disseminated yeast cells to invasive pseudohyphal and hyphal filaments leads to alterations in the gene expression patterns depending on the factors associated with the host niche. Moreover, in presence of solid biotic or abiotic substrates, such as tissues, prosthetics, or catheters, planktonic-yeast cells of *C. albicans* often adhere to surfaces to form a thick, three-dimensional community of yeast and filamentous cells called biofilms (5 – 8). More than one-fifth of all genes of the *C. albicans* genome show altered expression during this planktonic-biofilm growth phase transition (9). *C. albicans* biofilms become clinically relevant because of their multi-drug resistant nature (8). Drug resistance in biofilm cells is caused not only due to enclosure within an extracellular matrix but also by rewired metabolic state and elevated expression of genes for drug efflux pumps (9, 10). Since *C. albicans* is a major threat, especially in the case of immunocompromised individuals, it becomes clinically significant to determine the regulatory players bringing the gene expression modulations in this pathogenic fungus.

We previously identified a variant of histone H3, H3V^CTG^, as a negative regulator of biofilm growth (11). The binding of H3V^CTG^ nucleosomes at promoters of a subset of biofilm-relevant (BR) genes was found to be significantly higher as compared to the canonical histone H3 in cells grown in planktonic condition. Indeed, when cells were shifted from planktonic to biofilm mode of growth, the occupancy of H3V^CTG^ consequently reduced at these promoter regions. Furthermore, absence of H3V^CTG^ resulted in an altered expression of genes responsible for biofilm formation, even in planktonic cells (11). Enhanced binding of transcriptional modifiers at promoters of BR genes in planktonic cells was observed in absence of H3V^CTG^. These results helped us hypothesize that H3V^CTG^ favors the cells to maintain planktonic state by occluding the binding sites of transcriptional modifiers required for biofilm formation.

Previous studies highlighted the importance of nucleosome phasing in the context of gene regulation (12). At the eukaryotic promoters, a characteristic pattern of phased nucleosomes is observed with −1 nucleosome present before the transcription start site (TSS) followed by +1, +2, +3, etc., nucleosomes positioned after the TSS. The nucleosome-depleted region (NDR) is located between the −1 and +1 nucleosomes and serves as the site for recruitment of transcriptional machinery. It has been observed that the width of NDR is narrower and more confined during inactive transcription but expands and becomes wider during active transcription (13, 14). Thus, nucleosome positions are highly dynamic on chromatin (15). Their positions on DNA are adaptable depending on external cues and temporal gene expression. They have the propensity to slide along the DNA strands and can disassemble fully or partially. Moreover, histone proteins are subject to post-translational modifications (PTMs) which aid in the functional segregation of the genome (16). Nucleosome dynamics and thus chromatin plasticity enable the spatiotemporal regulation of gene expression and become an integral part of bringing about phenotypic alterations.

Nucleosomes are mapped to the genomic positions using multiple methods, most of which are based on digesting chromatin using micrococcal nuclease (MNase). MNase is both an endo- and exonuclease which can readily cleave free linker DNA but not the DNA sequences bound to a protein (17). Complete MNase digestion results in mononucleosomal length DNA fragments (~150 bp) that can be isolated and subjected to Next-Generation Sequencing (NGS) to identify genome-wide nucleosome positioning in a population of cells; this technique is known as MNase followed by high-throughput sequencing (MNase-seq) (18). Alternatively, techniques such as assay for transposase-accessible chromatin followed by high-throughput sequencing (ATAC-seq) assist in precisely predicting the open chromatin regions of the genome, not bound by histones or non-histone proteins (19). Briefly, the ATAC-seq method utilizes the genetically engineered hyperactive Tn5 transposase attached to NGS adapters. The Tn5 transposase cleaves the DNA fragments in an unbiased manner, simultaneously ligating adapter sequences. The process of concurrent adapter ligation followed by sequencing leads to the efficient detection of open chromatin regions and reduces loss in library complexity as compared to other methods. Moreover, the steric hindrance due to the enzyme’s molecular weight does not allow DNA to be cleaved at chromatin regions that are densely packed with proteins. Consequently, the reads originating from densely packed chromosomal regions are less abundant in ATAC-seq data as compared to a region with relatively open chromatin. Based on the experimental design, the downstream analysis of ATAC-seq data provides vital information about nucleosome-free regions (DNA fragment sizes < 100 bp) and nucleosome-occupied regions (DNA fragment sizes between 150 and 200 bp) associated with the open chromatin state. Henceforth, the complementation of MNase-seq and ATAC-seq methods provides a reliable overall view of chromatin landscape when cells are grown in a specific growth condition.

In this study, we sought to address the role of nucleosome occupancy in the context of planktonic to biofilm morphological transitions in *C. albicans*. High-throughput sequencing methodologies were used to determine the nucleosome occupancy, open chromatin regions, and gene expression alterations when cells were grown either in planktonic yeast form or induced to produce biofilms. We observed a significant reduction in the total number of nucleosomes, resulting in a more open chromatin landscape in biofilm cells compared to planktonic cells. Strikingly, we observed that gene regulation is modulated by the eviction of a previously identified histone H3 variant (H3V^CTG^), a repressor of biofilm growth in *C. albicans*.

## RESULTS

### Genome-wide occupancy of H3V^CTG^ at promoters of biofilm-relevant genes is higher in planktonic cells

**Fig 1:**
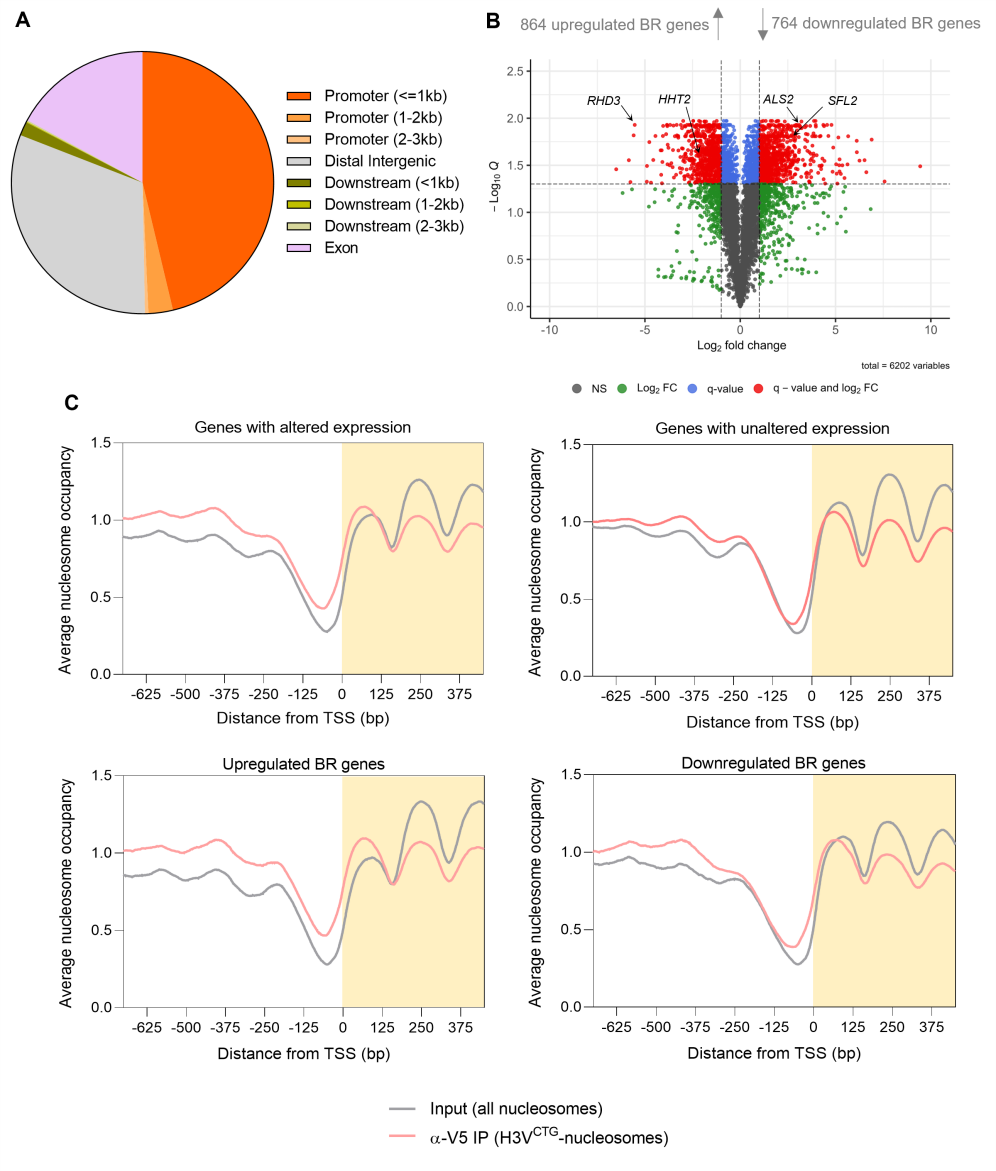
Genome-wide binding of H3V^CTG^ nucleosomes is enriched at promoters and upstream sequences of the TSS of BR genes in the planktonic cells. (**A**) Pie diagram representing the annotation analysis for H3V^CTG^ nucleosomes mapped to various regions of *C. albicans* genome. (**B**) Volcano plot depicting changes in the gene expression patterns based on the microarray data in biofilm cells as compared to cells grown in planktonic conditions. The *x*-axis indicates the log_2_(fold change) values, and the *y*-axis indicates the associated −log_10_(Q) values. The vertical and horizontal dotted lines indicate the cut-off applied for fold change and *Q*-values, respectively. Each dot on the graph represents the values corresponding to the individual gene of *C. albicans* genome. The red dots indicate the genes which are significantly different in expression and are either upregulated (upper right quadrant) or downregulated (upper left quadrant) in biofilm cells. Blue dots indicate the genes which are significantly different in their expression across the two growth conditions but do not pass the fold change cut-off. Green dots indicate the set of genes that are upregulated or downregulated based only on the fold change cut-off values but are not significantly different in their expression. Gray dots indicate the genes that neither pass the fold change nor the *Q*-value cut-off. FC, fold change; NS, non-significant. (**C**) Average nucleosome occupancy profiles for H3V^CTG^ nucleosomes (α-V5 IP) and all nucleosomes (input) are shown for genes that are altered (top, left) or unaltered (top, right) during planktonic to biofilm growth transition. Average nucleosome occupancy graphs were separately plotted for upregulated (bottom, left) and downregulated (bottom, right) BR genes. Nucleosome occupancy profiles are plotted against the distance from the TSS (0 bp). The shaded area of the graph represents the distance between the TSS and the ORF start site for >90% of the genes present in the *C. albicans* genome. IP, immunoprecipitated.

We previously reported the enhanced occupancy of H3V^CTG^ nucleosomes in planktonic cells as compared to H3 nucleosomes at promoters of a set of BR genes (11). To test this phenomenon genome wide, we performed MNase digestion followed by chromatin immunoprecipitation and next-generation sequencing (MNase ChIP-seq) for H3V^CTG^ by tagging both alleles of the *HHT1* gene with a V5-epitope in planktonic cells of *C. albicans* (Fig. S1A through C). Strikingly, the MNase ChIP-seq peak enrichment analysis showed enhanced binding of H3V^CTG^ at promoters of *C. albicans* genome (Fig. 1A; Table S1A and B in supplemental materials). To plot the average H3V^CTG^ occupancy at promoters, first, the TSS of genes present in the *C. albicans* genome was determined using a previous report (20). Subsequently, we determined the distance between the TSS and corresponding open reading frame (ORF) start site for each of these genes and plotted (Fig. S1D). The genome-wide average profile was indeed indicative of biased enrichment of H3V^CTG^ nucleosomes at promoters upstream of the TSS in planktonic cells (Fig. S1E).

To define the state of chromatin at promoters of BR genes, we performed the differential gene expression (DGE) analysis of two biological replicates of the wild-type *C. albicans* SC5314 strain grown in either planktonic or biofilm conditions (11). Approximately one-fourth of all *C. albicans* genes (1,628 genes) were found to have an altered expression based on log_2_(fold change) values of >1 and <−1, with a false discovery rate of 5% in our analysis (Fig. 1B). Out of these altered genes, 864 genes were found to be upregulated, and 764 genes were found to be downregulated when cells were shifted from planktonic to biofilm conditions (Table S2 in supplemental materials). Functionally verified genes such as *ACE2*, *ZCF8*, and *CRZ2* which are responsible for *C. albicans* adherence to a substrate and are known to be expressed in biofilm cells (21) were found to be upregulated in the DGE analysis. Other genes required for *C. albicans* filamentation (*SLF2*, and *CPH1*) (22, 23), cell-cell adhesion (*ALS2*) (24), and extracellular proteolytic activity (*SAP1*, *SAP5*, and *SAP10*) (25, 26) having an established role in biofilm formation were also found to be upregulated. Among the differentially downregulated genes, we observed the enrichment of genes repressed during hyphal initiation and biofilm formation such as *NRG1*, *RHD3*, and *TPK1* (27 – 29). Strikingly, we also observed the downregulation of genes responsible for coding the canonical (H2A, H2B, H3, and H4) and variant (H3V^CTG^ and Cse4) histone proteins in *C. albicans*.

To determine the genome-wide occupancy of H3V^CTG^ in the planktonic cells and its effect on gene expression, we generated average nucleosome occupancy profiles focusing on the promoter regions of BR genes. Since the TSS data were only available for 737 upregulated BR genes and 694 downregulated BR genes, we performed further analysis using this set of 1,431 out of 1,628 differentially regulated genes. Remarkably, biased enrichment of H3V^CTG^ nucleosomes was evident at promoters of genes with altered expression across the two conditions as compared to genes with unaltered expression (Fig. 1C; Fig. S2). These differences were more enhanced when we separately examined the average H3V^CTG^-nucleosome occupancy at genes upregulated and downregulated in biofilm cells (Fig. 1C). The +1 nucleosome in upregulated BR genes in planktonic cells was occupied by H3V^CTG^ with a narrow NDR upstream of the TSS, as compared to genes downregulated in biofilm cells. Thus, we identify a molecular switch regulated by biased genome-wide occupancy of H3V^CTG^ nucleosomes at promoters of BR genes to determine the planktonic or biofilm mode of growth in *C. albicans*.

### H3V^CTG^ nucleosomes in planktonic cells are devoid of transcriptionally active marks

**Fig 2:**
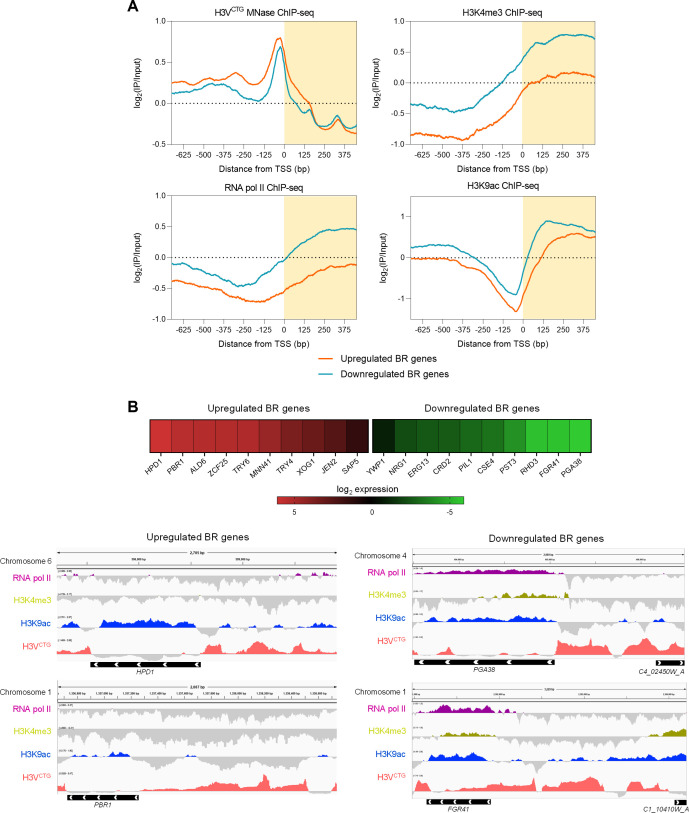
Regions associated with transcriptionally active histone H3 post-translational marks and H3V^CTG^ nucleosomes are mutually exclusive at promoters of BR genes in planktonic cells. (**A**) Average enrichment profiles are plotted for H3V^CTG^ (top left), H3K4me3 (top right), RNA pol II (bottom left), and H3K9ac (bottom right) obtained from ChIP-seq data for differentially expressed BR genes. The average profile in each condition is plotted as a measure of distance from the TSS (0 bp), and IP samples are normalized to the input from individual ChIP-seq experiments. The orange and blue profiles are generated for genes upregulated and downregulated, respectively, in biofilm cells as compared to planktonic cells. IP, immunoprecipitated. (**B**) Top, heat map representing the expression of BR genes in biofilm cells compared to planktonic cells. The expression of genes is plotted based on the log_2_ fold change values from the microarray performed from *C. albicans* planktonic and biofilm cells. Bottom, representative snapshots depicting the binding of RNA pol II, H3K4me3, H3K9ac, and H3V^CTG^ at promoters of upregulated and downregulated BR genes. The histograms represent the log_2_ ratio for IP/input for each ChIP-seq data set. Black bars represent the genes, and the white arrowheads demarcate the direction of transcription. Numbers represent the chromosomal coordinates.

Our previous analysis indicated that H3V^CTG^ nucleosomes are enriched at promoters of BR genes in planktonic cells. Consequently, it prompted us to compare the H3V^CTG^ occupancy with histone H3 PTMs associated with actively transcribing regions of the *C. albicans’* genome. We used previously published ChIP-seq data sets for histone H3 tri-methylated at the fourth lysine residue (H3K4me3) and acetylated at the ninth lysine residue (H3K9ac) for this analysis along with RNA polymerase II (RNA pol II) (30). To determine the genome-wide patterns and confirm the enrichment of histone H3 PTMs at promoters of actively transcribing genes, we equally divided *C. albicans* genes based on transcriptome data available for planktonic cells (11) into three categories: highly transcribing, moderately transcribing, and lowly transcribing. We observed that the extent of enrichment of H3K4me3 and H3K9ac marks is highest among the highly transcribing genes and decreases gradually in moderately and lowly transcribing genes (Fig. S3A). RNA pol II ChIP-seq profiles matched this observation. Strikingly, we observed that the biased enrichment of H3V^CTG^ at promoters of BR genes was mutually exclusive to transcriptionally active histone H3 PTMs (Fig. 2A). Genes upregulated during biofilm condition did not show enrichment of either of the histone H3 PTMs at promoters when compared to the downregulated BR genes. (Fig. 2B; Fig. S3B). In conclusion, our analysis reveals that H3V^CTG^ nucleosomes in planktonic cells are not modified post-translationally with histone H3 PTMs associated with active transcription to keep the biofilm circuitry turned off.

### An open chromatin state is achieved when *C. albicans* cells are shifted from planktonic to biofilm mode of growth

To delineate the role of nucleosomes and their association with alterations in gene expression patterns in planktonic to biofilm growth transition, we first defined the genome-wide nucleosome positions in planktonic and biofilm cells. Nucleosomes can be mapped to the genomic positions by digesting chromatin using MNase-seq (31). We used a previously published MNase-seq data set where wild-type *C. albicans* SC5314 cells were grown under planktonic conditions (32). The analysis for MNase-seq reads was performed, and nucleosome peaks were called using the Bioconductor package “nucleR” (33) (Fig. S4A). A total of 96,699 nucleosome peaks were obtained from the analysis for cells grown in the planktonic condition (Table S3A). The previously published MNase-seq protocol (32) was further extended to wild-type *C. albicans* SC5314 cells grown in biofilm condition for 48 h at 37°C. *In situ* MNase digestion was performed on nuclei isolated from biofilm-induced cells which yielded 49,078 nucleosome peaks (Table S3B). Thus, from our analysis, we observed a significant reduction in the number of nucleosomes when cells were shifted from planktonic to biofilm conditions. This was in corroboration with our previous results where we observed downregulation of histone proteins in biofilm cells. The presence of nucleosomes was additionally confirmed at an intergenic locus for planktonic and biofilm cells (Fig. S4B and C). To further determine the pattern of nucleosome organization at promoters of all the genes in *C. albicans*, we determined the average nucleosome occupancy profiles and plotted them as the distance from the TSS for cells grown in both conditions (Fig. S4D). We observed that cells maintained the typical pattern of nucleosome organization at eukaryotic promoter regions with a wide NDR proximal to the TSS, flanked by nucleosome-occupied regions. However, the nucleosome phasing was altered in cells grown in the biofilm condition at the +1 nucleosome position when compared to the planktonic grown cells.

**Fig 3:**
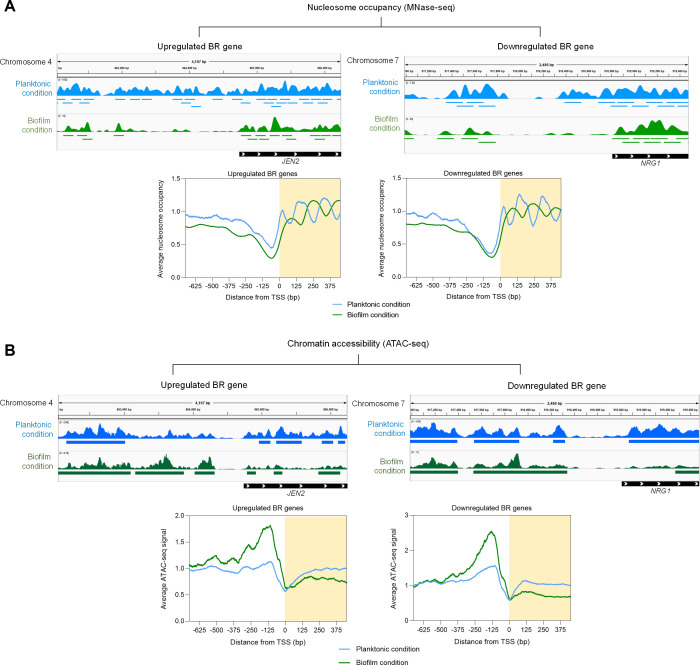
Nucleosome depletion in biofilm cells results in an open chromatin state at promoters of BR genes. (**A**) Top, genome browser snapshot for genes *JEN2* (upregulated BR gene) and *NRG1* (downregulated BR gene) showing the reduction in nucleosome-occupied regions at the respective gene promoters in biofilm cells. The blue and green bars below each histogram in the genome browser snapshots represent nucleosome-occupied regions in individual growth conditions, whereas the black bars indicate the genes with white arrowheads demarcating the direction of transcription. Numbers represent chromosomal coordinates. Bottom, average nucleosome occupancy plots showing the nucleosome depletion at promoters of all upregulated and downregulated BR genes. (**B**) Top, genome browser snapshot for genes *JEN2* and *NRG1* showing increased chromatin accessibility at promoters in biofilm cells. The accessible chromatin sites are shown by blue and green bars below the histograms (Table S3C and D in supplemental materials), while the genes are represented by black bars with white arrowheads denoting transcriptional direction. The chromosomal coordinates are represented by the numbers. Bottom, average ATAC-seq signals depict increased chromatin accessibility even at promoters of downregulated BR genes in biofilm cells. All the graphs are plotted as a measure of distance from the TSS for genes upregulated or downregulated during planktonic (shown in blue) to biofilm (shown in green) growth transition. The shaded area of the graphs represents distance between the TSS and the ORF start site for >90% of genes present in the *C. albicans* genome.

Since the above results suggested a global reduction in the total number of nucleosomes when cells were shifted from planktonic to biofilm mode of growth, we tested the state of chromatin at the TSS by employing the ATAC-seq method. Average ATAC-seq signal profiles were generated from the fragment size <100 bp for three classes of genes (highly, moderately, and lowly expressed genes), which further confirmed that promoter regions are largely devoid of nucleosomes at varying extent (Fig. S4E). As expected, the genes with the maximum transcription rate produced the highest ATAC-seq signals from promoters, followed by moderately and lowly transcribing genes both in planktonic and biofilm cells (Fig. S4E). Thus, to delineate the role of nucleosome positioning in the regulation of gene expression when cells are shifted to biofilm conditions, we compared chromatin organization at promoters of differentially expressed genes across the two modes of growth (Fig. S4F). Nucleosome occupancy at promoters of upregulated BR genes was found to be higher in planktonic cells and decreased when cells were induced to form biofilms (Fig. 3A). A similar decrease in nucleosome occupancy was observed for genes that are downregulated in biofilm condition suggesting open chromatin regions associated even with a transcriptionally repressed state. To confirm this unusual observation, we complemented the MNase-seq data set with ATAC-seq (DNA fragments <100 bp) and scanned promoters of differentially expressed genes for open chromatin regions (Fig. S4F). Strikingly, we observed open chromatin regions devoid of nucleosomes even at promoters of genes downregulated in biofilm cells, while an expected increase in the open chromatin state was found for upregulated BR genes (Fig. 3B; Fig. S5 and S6). Thus, our results confirm the presence of an atypical nucleosome organization at promoters of BR genes when cells are shifted from planktonic to biofilm mode of growth. This uncharacteristic chromatin feature at promoters of downregulated BR genes can be correlated with an overall decrease in the levels of histone proteins (Fig. 1B).

### Nucleosome depletion in biofilm cells promotes the accessibility of transcription modifiers

**Fig 4:**
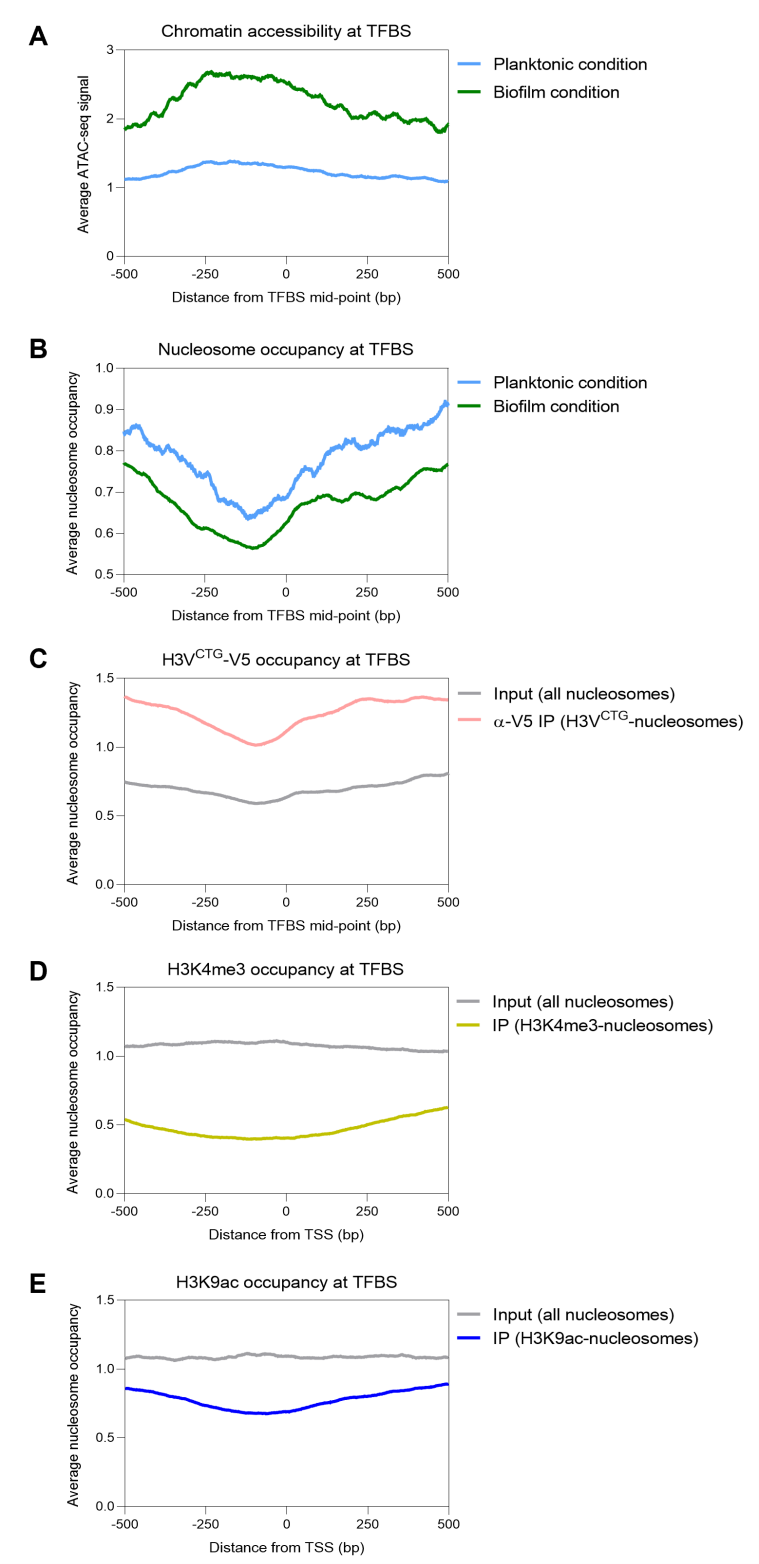
A nucleosome-depleted, open chromatin state facilitates access to transcription modulators in biofilm cells. (**A**) Average nucleosome occupancy and (**B**) chromatin accessibility plots are shown for the binding of master regulators of biofilm formation (Bcr1, Tec1, Ndt80, Efg1, Rob1, and Brg1) in planktonic (blue) and biofilm (green) cells. (**C**) Average nucleosome occupancy at the known TFBS was plotted for (**C**) H3V^CTG^ nucleosomes, (**D**) H3K4me3 nucleosomes, and (**E**) H3K9ac nucleosomes for planktonic cells. The average profiles were generated for ±500 bp from the TFBS midpoint (0 bp).

Nucleosomes act as barriers for chromatin modifiers by occupying the sites of transcription factor binding. Moreover, the literature provides evidence for a well-orchestrated biofilm gene circuitry of *C. albicans* modulated by nine master regulators, viz., Bcr1, Tec1, Ndt80, Efg1, Rob1, Brg1, Gal4, Rfx1, and Flo8 (9, 34). The absence of these master regulators, barring Gal4, leads to defective biofilm formation in biofilm-inducing conditions. Thus, to determine whether presence of nucleosomes occludes the transcription factor binding sites (TFBS) across the two modes of *C. albicans* growth, we obtained the data for binding sites of six of these master regulators in biofilm cells from a previously published report (9). The average ATAC-seq signals for a total of 1786 TFBS were plotted, and we observed an increase in the open chromatin region in biofilm cells as compared to planktonic (Fig. 4A). Furthermore, nucleosome occupancy was decreased at the TFBS in biofilm cells paving way for transcription modulators to access chromatin (Fig. 4B). Previously, it was also shown that binding of H3V^CTG^ at promoter regions in planktonic cells restricts the access of Bcr1 (11). The absence of H3V^CTG^ in planktonic cells led to increased binding of Bcr1 at these promoter regions. Therefore, we examined the binding sites of six master regulators (Bcr1, Tec1, Ndt80, Rob1, Efg1, and Brg1) for presence of H3V^CTG^ nucleosomes and observed an increased deposition of H3V^CTG^ at these TFBS in planktonic cells (Fig. 4C). We additionally found depletion of transcriptionally active histone marks (H3K4me3 and H3K9ac) at these TFBS in planktonic cells (Fig. 4D and E). Altogether, our results confirm the presence of an open chromatin state and decreased nucleosome occupancy leading to efficient gene regulation via transcriptional modifiers (activators and repressors) in biofilm mode of growth.

### Biased eviction H3V^CTG^ nucleosomes occurs from promoters of *C. albicans* genes during planktonic to biofilm transition

**Fig 5:**
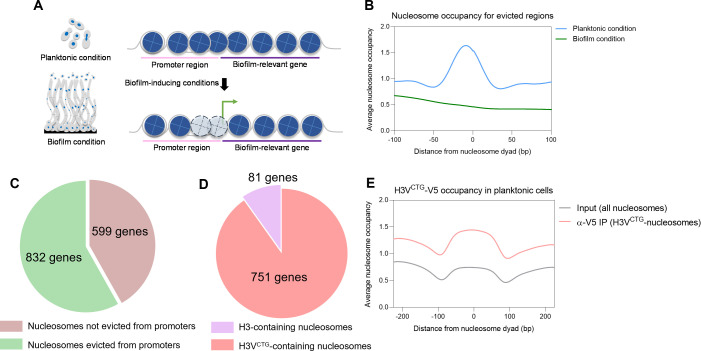
Biased eviction of H3V^CTG^-nucleosomes from promoters of BR genes. (**A**) Schematic representing the nucleosome eviction from promoters of BR genes when cells are grown in planktonic conditions as compared to biofilm conditions. Nucleosomes that are evicted in biofilm cells are marked with faded color and dashed outlines. (**B**) Average nucleosome occupancy was mapped for nucleosomes evicted from promoters of all genes in planktonic (blue) or biofilm (green) cells. The *x*-axis indicates the distance from the nucleosome dyad (0 bp), and the *y*-axis represents the average occupancy. (**C**) Pie diagram depicting the fraction of genes with and without nucleosomes evicted from promoters of BR genes during planktonic to biofilm growth transition. (**D**) Pie diagram representing the fraction of genes with H3 and H3V^CTG^ nucleosomes evicted from promoters of BR genes. (**E**) Average nucleosome occupancy profile of H3V^CTG^ nucleosomes at regions of nucleosome eviction during the biofilm mode of growth. The input and H3V^CTG^-IP samples are color coded as shown and plotted as a measure of distance from the nucleosome dyad (0 bp). IP, immunoprecipitated.

The transcriptome analysis of cells grown in planktonic and biofilm conditions indicated downregulation of histone proteins, which was in accordance with the lesser number of nucleosomes present in biofilm cells. This prompted us to examine whether nucleosome eviction occurs preferentially from promoters of BR genes in biofilm cells. For this, nucleosomes were compared across the two modes of growth, and nucleosomes present exclusively in planktonic cells were defined. Lack of a concurrent nucleosome in biofilm cells indicated that nucleosomes were displaced or removed from those sites in biofilm cells (Fig. 5A). We identified 25,046 distinct nucleosomes in planktonic cells that are absent in biofilm cells and confirmed nucleosome eviction at those sites by plotting the average occupancy profiles in planktonic and biofilm cells (Fig. 5B). Our findings revealed that a significant proportion of BR genes exhibited the removal of nucleosomes from their promoter regions (Fig. 5C). Among the differentially expressed genes, the majority of genes that displayed nucleosomes eviction from their promoters were upregulated in biofilm cells (471 genes out of 737 genes). Surprisingly, more than half of downregulated BR genes (361 genes out of 694 genes) also exhibited a similar trend of nucleosome eviction. To investigate whether the evicted nucleosomes at promoters of BR genes contained H3V^CTG^, we performed an overlap analysis between unique nucleosome peaks in planktonic cells and those identified through the nucleR analysis of H3V^CTG^ MNase ChIP-seq (Table S1C in supplemental materials). Our results provide strong evidence for eviction of H3V^CTG^ nucleosomes at promoter regions of approximately 90% (751 of 832 genes) of BR genes that undergo nucleosome eviction (Fig. 5D). Specifically, we observed the presence of evicted H3V^CTG^ nucleosomes in 428 out of 471 upregulated BR genes and 323 out of 361 downregulated BR genes. We additionally observed an enhanced occupancy of H3V^CTG^ nucleosomes at the evicted regions in planktonic cells (Fig. 5E). Taken together, our observations establish that eviction of H3V^CTG^ nucleosomes at promoters of a majority of BR genes, whether upregulated or downregulated, is the mode of transcriptional regulation in biofilm cells.

## DISCUSSION

In this study, we performed genome-wide identification of H3V^CTG^ nucleosomes in *C. albicans* planktonic cells, providing evidence of their enhanced presence at promoters of BR genes. The H3V^CTG^ nucleosomes lacked transcriptionally active histone H3 PTMs (H3K4me3 and H3K9ac), further exerting its previously established role as the negative regulator of biofilm formation. By integrating the multi-omics approach, we investigated the impact of nucleosome positioning on gene expression modulation upon biofilm induction and characterized the chromatin architecture at promoters of BR genes. Our findings revealed a decrease in nucleosome occupancy and presence of an open chromatin state at promoters of BR genes in biofilm cells. Importantly, we demonstrated that the increased chromatin accessibility facilitates binding of transcriptional modifiers (activators and repressors), thus exerting control over gene expression. Additionally, we observed preferential eviction of H3V^CTG^ nucleosomes from promoters of BR genes in biofilm cells. Altogether, we uncovered the mechanism by which H3V^CTG^ helps in achieving a specific growth mode, whether planktonic or biofilm state, by its preferential binding at promoters of BR genes.

Nucleosome organization and transcription factor binding at promoters are the key determinants of gene expression, influenced by intrinsic cues from the extracellular environment (12). Specific transcription factors, such as Bcr1, Tec1, Ndt80, Efg1, Rob1, Brg1, Gal4, Rfx1, and Flo8, have been identified as master regulators that orchestrate the gene circuitry required for successful biofilm establishment in *C. albicans* (9, 34). It has been shown previously that deletion mutants of these master regulators can grow in planktonic conditions but fail to form robust *C. albicans* biofilms, except for Gal4 (9, 34). Strikingly, enhanced biofilm formation was observed in the deletion mutants of three (Bcr1, Tec1, and Ndt80) out of the six master regulators (Bcr1, Tec1, Ndt80, Efg1, Rob1, and Brg1) upon simultaneous deletion of the *HHT1* gene that codes for H3V^CTG^ (11). This shows that H3V^CTG^ blocks the sites of transcription modifiers even in biofilm cells, and in the absence of H3V^CTG^, those sites become accessible to them.

**Fig 6:**
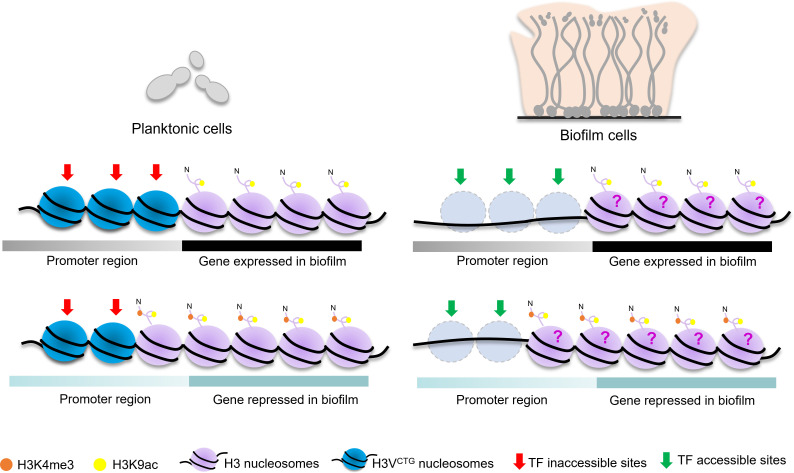
A proposed model by which biased eviction of H3V^CTG^ nucleosomes leads to differential gene expression in biofilm cells. Schematics represent the involvement of H3V^CTG^ in forming a repressive chromatin state for BR genes. During the planktonic mode of growth, H3V^CTG^ nucleosomes are preferentially bound at promoters of BR genes, are largely devoid of transcriptionally active histone marks (H3K4me3 and H3K9ac), and prevent transcription modifiers from occupying their binding sites. Therefore, chromatin enriched with H3V^CTG^ ensures restricted access of transcriptional modulators to promoters of BR genes in planktonic cells resulting in a turned-off state of the biofilm gene circuit. On the other hand, in biofilm cells, when nucleosomes carrying H3V^CTG^ are evicted, an open chromatin state is established at promoters of BR genes thereby allowing the enhanced binding of transcriptional activators and repressors, leading to altered gene expression patterns favoring robust biofilm growth. This is a classic example of growth form transitions of a unicellular organism modulated at the chromatin level by a histone H3 variant. Future investigations of PTMs of both histone H3 and H3V^CTG^ will shed light on the roles of these epigenetic marks in regulating the gene expression patterns during planktonic to biofilm growth transition. TF, transcription factor.

In summary, our study adds to the existing knowledge of the intricate and dynamic nature of transcriptional regulation in *C. albicans* biofilm formation. The open architecture of chromatin facilitated by the eviction of H3V^CTG^ nucleosomes during biofilm formation aids in modulating the gene expression (Fig. 6). This process influences not only the upregulated BR genes but also those that are downregulated in biofilm cells, emphasizing the significant role of transcriptional repressors in regulating *C. albicans* biofilms. Future investigations leading to determination of factors responsible for preferential incorporation of H3V^CTG^ into chromatin and subsequent eviction would open new avenues for developing intervention strategies for biofilm formation.

## MATERIALS AND METHODS

### Strains, media, and growth conditions

All *C. albicans* strains used in this study (listed in Table S4A) were routinely grown in 1% yeast extract, 2% peptone, 2% dextrose, and 0.1 µg/mL uridine (YPDU) at 30°C.

### Construction of *C. albicans* H3V^CTG^-V5 tagged strain

The C-terminal of H3V^CTG^ (*HHT1* or *C3_07090W_A*) was amplified using 6791V5-CFP and 6791V5-CRP primers and cloned in pBS-NAT (35) plasmid at SacI and SacII sites resulting in pRA01 plasmid. Transformation of *C. albicans* strain LR144 (*HHT1/HHT1-V5-HIS*) (11) was performed by amplifying the *HHT1-V5-NAT* fragment from pRA01 using primer pairs 6791V52LFP and RA1. Transformants were selected for nourseothricin (100 µg/mL) resistance and confirmed by Southern blotting. Oligos used for strain construction are listed in Table S4B.

### Genomic DNA isolation from *C. albicans*

Ten-milliliter culture of *C. albicans* cells was grown overnight in YPD and harvested by centrifugation at 3,800 rpm for 5 min. Cells were washed with autoclaved water and resuspended in 200-µL extraction buffer. Cells were lysed by vortexing with 0.3 g of acid-washed glass beads and 200 µL of phenol:chloroform:isoamyl alcohol (25:24:1). Next, 200 µL of 1× Tris-EDTA (TE) solution was added to the tubes followed by centrifugation at 13,000 rpm for 10 min. The supernatant was collected, and DNA was precipitated by 100% ethanol at −20°C for 1 h. The tubes were centrifuged at 13,000 rpm, and the pellet obtained was washed with 70% ethanol. The pellet was air dried and resuspended in 30 µL of 1× TE and 1-µL RNase A (10 mg/mL).

### Western blotting

*C. albicans* strains were grown overnight in YPD, and 6 × 10^7^ cells were harvested by centrifugation at 3,800 rpm for 5 min. Cells were washed with 1 mL of autoclaved water and resuspended in 400 µL of 12.5% trichloroacetic acid solution. The cell suspension was frozen at −20°C overnight. Next day, the cells were thawed on ice and pelleted at 13,000 rpm, 4°C for 10 min. The pellet was washed in 80% acetone solution followed by pelleting at 13,000 rpm, 4°C for 10 min. After two rounds of washing, the pellet was air dried and resuspended in 50 µL of lysis buffer (1% SDS + 0.1 N NaOH) with 1× loading dye. The lysate was incubated in a boiling water bath for 10 min. Proteins were separated by electrophoresis on a 12% SDS-PAGE gel and blotted onto a nitrocellulose membrane (Amersham Protran Premium, catalog number 10600003) in a semi-dry apparatus (Bio-Rad). The blotted membranes were blocked by 5% skim milk containing PBS (pH 7.4) for 30 min at room temperature. The membranes were then incubated with 1:5,000 dilution of anti-V5-antibodies (Invitrogen, catalog number R6025) or 1:5,000 dilution of anti-PSTAIRE antibody (Abcam, catalog number ab10345) in 2.5% skim milk in PBS at 4°C overnight with constant shaking. Next, the membrane was washed thrice with PBST (0.1% Tween-20 in 1× PBS) solution. Anti-mouse HRP-conjugated antibodies (Bangalore Genei) were added at a dilution of 1:5,000 in 2.5% skim milk PBS solution and incubated for 1–2 h at 4°C on constant shaking. The membranes were then washed thrice with the PBST solution. Signals were detected using the chemiluminescence method.

### Southern blotting

RA107 strains were confirmed using Southern hybridization. Genomic DNA was isolated and quantified using Quantity One software (Bio-Rad). Six to eight micrograms of DNA was digested and separated on an agarose gel. The gel was sequentially treated with 0.5 N HCl for acid nicking of DNA at room temperature for 10 min. This was followed by denaturation and neutralization of DNA using denaturation buffer (0.5 N NaOH and 1.5 N NaCl) and neutralization buffer (1.5 M Tris-Cl, pH 8.0, and 1.5 N NaCl), respectively, for 30 min each at room temperature. Acid-nicked DNA was transferred by capillary action onto a positively charged nylon membrane (Amersham Hybond-N+, catalog number RPN303B) in 10× saline-sodium citrate (SSC) buffer at room temperature for 12–16 h. Following transfer, the membrane was washed with 2× SSC for 15 min, dried, and crosslinked by exposure to UV for 5 min in Genelinker (Bio-Rad, 12,000 μJ × 100). The blot was pre-hybridized in 10-mL 1× Southern hybridization buffer (10 g SDS, 5.8 g NaCl, 2.4 mL 0.5 M EDTA, 20 mL 1 M sodium phosphate, pH 7, for 200 mL) at 65°C for 2–4 h. The specific probe amplified using 6791SUSFP/6791USNAT1RP primers was radiolabeled with ^α32^P-dCTP using a random primer labeling kit (BRIT-LCK-102). Fifty-nanogram probe DNA in an appropriate volume of autoclaved water was boiled for 5 min and chilled immediately. Next, 5-µL random primer buffer, 5-µL random primer, 12-µL dNTP cocktail except dCTP, 4-µL ^α32^P-dCTP, and 2-µL Klenow polymerase were added to the reaction mixture followed by incubation at 37°C for 40 min. The reaction was stopped by 2-µL 0.5 M EDTA, and volume was made up to 100 µL by adding 1× TE solution. The pre-hybridized blot was then incubated with the radiolabeled probe for 16 h at 65°C. The blot was washed by wash buffer I (2× SSC, 1% SDS) twice for 30 min each at 65°C and wash buffer II (0.5× SSC, 0.1% SDS) thrice for 20 min each at 65°C. The membrane was exposed to Phosphor imager film, and the image was captured by Phosphorimager.

### Quantitative PCR

Primers designed for quantitative PCRs (qPCRs) (listed in Table S4B) amplified gene products between 100 and 120 bp long. Analysis of the melt curve was performed to ensure specific amplification without any secondary non-specific amplicon generation. The nucleosome positions in planktonic and biofilm conditions were confirmed using “In” (RS219 and RS220) primers and “Out” (RS217 and RS218) primers. PCR was carried out in the final volume of 10 µL using 2× Sensi fast SYBR mix (BIO-98020). Real-time PCR was carried out in CFX96 Touch Real-Time PCR Detection System using the following reaction conditions: 95°C for 2 min, 95°C for 30 s, 55°C for 30 s, and 72°C for 30 s for 40 cycles.

### Biofilm assay

For the growth of cells in biofilm condition, a single colony was inoculated in 5-mL YPDU containing 50 mM galactose and incubated overnight at 30°C. Biofilm was grown on a six-well polystyrene plate. The wells were coated with 1 mL fetal bovine serum (FBS) for 4–12 h before the experiment and washed with 2-mL autoclaved 1× PBS solution. A total of 0.5 OD equivalent cells/mL were added from the overnight culture, and the volume was made up to 3 mL with YPDU media. The polystyrene plate was incubated at 37°C with agitation at 100 rpm for a period of 1.5 h (adherence step). Floating cells and excess media were removed, and wells were washed with 1× PBS solution. Three milliliters of fresh YPDU media was added to the wells and incubated at 37°C for 48 h at 40 rpm agitation (maturation step). The biofilm cells were scrapped, and the content of each well was transferred to a 50-mL tube for downstream processing of the samples.

### Nuclei isolation

For nuclei isolation from *C. albicans* cells, 100 OD equivalent cells were grown in planktonic and biofilm conditions and harvested. Cells were crosslinked with formaldehyde (1% final concentration) for 15 min at room temperature (RT). The reaction was stopped using 125 mM glycine and incubated at RT for 5 min. Fifty OD cells were washed with autoclaved water and treated with resuspension buffer (0.5-mL β-mercaptoethanol and 9.5-mL water) at 30°C for 30 min at 180 rpm. Cells were washed once with 5-mL S-buffer (1.2 M Sorbitol and 20 mM Na-PIPES, pH 7.4) and resuspended in the same buffer. A 10 µg/mL of Zymoylase 20T (MP Biomedicals, catalog number 320921) was used for spheroplasting the cells and incubated at 30°C at 60 rpm for 45 min for planktonic cultures and 90 min for cells grown in the biofilm condition. After 90% spheroplasting was achieved, spheroplasts were washed twice with ice-cold 5-mL S-buffer at 4,000 rpm at 4°C. Ten milliliters of lysis buffer [18% Ficoll 400, 20 mM Na-PIPES (pH 7.4), 0.5 mM MgCl_2_, and 0.1 mM PMSF] was mixed thoroughly at high speed using a magnetic bead for 5 min at 4°C. Out of the 10-mL lysis buffer, 2 mL was used to resuspend the spheroplasts. Clumps were carefully removed to make the suspension consistent. Spheroplasts were poured into the remaining lysis buffer in a drop-wise manner, and stirring was performed at a minimal speed for 15 min at 4°C. The suspension was poured into a fresh 50-mL falcon and gently vortexed for 3 min at 4°C. Glycerol-Ficoll cushion [20% glycerol, 8% Ficoll 400, 20 mM Na-PIPES (pH 7.4), 0.5 mM MgCl_2_, and 0.1 mM PMSF] was prepared and poured into a 50-mL oakridge tube. This was pre-chilled before usage. The spheroplasts were laid on top of the Glycerol-Ficoll cushion and centrifuged at 12,500 rpm for 40 min at 4°C. The supernatant was gently decanted, and the pellet (nuclei) fraction was gently resuspended in 1-mL ice-cold PC buffer [20 mM Na-PIPES (pH 7.4), 2 mM CaCl_2_, 0.5 mM MgCl_2_, 1 mM PMSF, and 1× Protease Inhibitor Cocktail].

### MNase digestion and chromatin extraction

MNase digestion and chromatin extraction were performed as previously described (32) with some modifications. Total nuclei preparation was pre-warmed at 37°C for 3 min. A 100-µL aliquot of the nuclei preparation, which was used as genomic DNA control. It was treated similarly to the rest of the sample except for the enzymatic digestion. In the remaining nuclei preparation, micrococcal nuclease (NEB catalog number M0247S) was added to the final concentration equivalent to 2,000 gel units and incubated at 37°C for 20 min, with intermittent gentle shaking. The reaction was stopped using ethylene glycol-bis(β-aminoethyl ether)-N,N,N′,N′-tetra acetic acid (EGTA) and ethylenediamine tetraacetate (EDTA) to a final concentration of 2 mM. One percent sodium dodecyl sulfate (SDS) was added to break the nuclear membrane to obtain the chromatin fraction. This suspension was incubated on ice for 15 min and centrifuged at 14,000 rpm for 20 min at 4°C. The supernatant was aliquoted in a fresh 1.5-mL microfuge tube, and the pellet was discarded. The reverse crosslinking step was performed by one-fourth volume of 5 M NaCl in elution buffer (0.1 M NaHCO_3_ and 1% SDS) and incubating at 65°C overnight. This was followed by Proteinase K treatment by adding 10 mM EDTA (pH 8.0), 4 mM Tris-Cl buffer (pH 6.8), and 2 µL of 20 mg/mL of Proteinase K enzyme. Incubation was performed at 37°C for 2 h. Phenol:chloroform:isoamyl alcohol was added to the solution in a 1:1 ratio and vortexed vigorously, followed by centrifugation at 13,500 rpm for 15 min at RT. The aqueous layer was gently aspirated in a fresh microfuge tube. This was followed by DNA precipitation by adding 1 µL of glycogen, 70 µL of 3 M Na-acetate, and one volume of absolute ethanol and stored at −20°C overnight. Centrifugation was performed at 14,000 rpm for 30 min at 4°C. The DNA pellet was washed using 70% ethanol and resuspended in 18 µL of water. Two microliters of RNase A (10 mg/mL) was added for degrading the RNA molecules, and the chromatin integrity was checked on 2% agarose gel. The isolated DNA fragments were then used for semi-quantitative and qPCR analysis.

### Chromatin immunoprecipitation

For the chromatin immunoprecipitation (ChIP) assay, the nuclei were isolated, and MNase digestion was performed as mentioned previously. The MNase reaction was stopped using 2 mM of EGTA and 2 mM EDTA, and the reaction mix was incubated on ice for 15 min. The chromatin extraction was carried out by passing the MNase-digested nuclei fraction through a 24-gauge needle five times and a 30-gauge needle three times. This was followed by centrifugation at 13,000 rpm for 30 min at 4°C. The supernatant was aliquoted in a fresh microfuge tube, and 100 µL of the fraction was aliquoted as the input sample (processing for de-crosslinking mentioned below). The rest of the sample was divided into two fractions: IP (+ antibody) and mock (− antibody). To the IP fraction, 3 µL of anti-V5 antibody (Invitrogen, catalog number R960-25) was added, and the tubes were incubated at 4°C overnight on rotation. A 30-µL of Protein-A beads (Sigma, catalog number P3391) was added to IP and mock samples and incubated again at 4°C for 8–12 h on rotation. The beads were then washed twice with 1 mL each of low salt, high salt, LiCl wash buffers, and 1× TE solution. The samples were rotated in a rotaspin for 15 min at RT for every wash. After washing, the DNA was eluted from the beads twice using 250 µL of elution buffer. The samples for elution were incubated at 65°C for 5 min, rotated for 15 min at RT, and collected by centrifugation. The samples were processed for de-crosslinking by adding 20 µL of 5 M NaCl in the elution buffer to each tube and incubated at 65°C overnight. This was followed by the removal of proteins by adding 10 µL of 0.5 M EDTA, 20 µL of 1 M Tris-Cl (pH 6.8), and 2 µL of Proteinase K (20 mg/mL). This reaction mix was incubated at 37°C for 2 h. After incubation, samples were treated with an equal volume of phenol-chloroform-isoamyl alcohol (25:24:1) mix, and the aqueous phase was extracted by centrifugation. DNA was precipitated by the addition of one-tenth volume of 3 M Na-acetate, 1-µL glycogen (Sigma, 10 mg/mL), and 1-mL absolute ethanol and incubated at −20°C for at least 12 h. The samples were centrifuged at 14,000 rpm for 45 min at 4°C followed by washing with ice-cold 70% ethanol. Seventy percent ethanol was decanted, and the DNA pellets were air dried. The input sample was resuspended in 25 µL of autoclaved MilliQ water and 5 µL of 10 mg/mL RNase A. The resuspended input sample was incubated at 37°C for 15 min before usage. The IP samples were resuspended in 20-µL MilliQ water. The ChIP was confirmed using RS219/RS220 primers and sent for sequencing.

### ATAC assay

For the ATAC assay, the protocol was modified from the published literature (36, 37). Nuclei isolation was performed for *C. albicans* wild-type cells SC5314 grown in planktonic and biofilm conditions. The isolated nuclei were counted using a hemocytometer, and 10^5^ nuclei equivalent were spun down at 4,000 rpm for 5 min at 4°C. The supernatant was carefully removed using a pipette, and the nuclei pellet was resuspended in transposition mix as follows: 5 µL of 2× TD Tagment DNA buffer (Illumina, catalog number 15027866), 1 µL of TDE1 enzyme (Illumina, catalog number 15027865), and 4 µL of water. The tagmentation reaction was carried out for 60 min at 37°C, and 10 µL of water was added post-incubation. For enrichment of tagmented DNA, PCR was set using 27.5 µL of KAPA2G Robust HotStart ReadyMix (catalog number KK5701) and 2.5 µL of 25 µM each of Ad1_moMX/Ad2.1 primer pair. The PCR was carried out for seven cycles as follows: 72°C for 5 min, 98°C for 30 s, 98°C for 10 s, 63°C for 30 s, and 72°C for 60 s. The enriched tagmented DNA was size selected in two steps for <600 bp and >50 bp fragments using AMPure SPRI beads (Beckman Coulter, catalog number A63881). The DNA fragments were finally eluted in 20-µL 10 mM Tris-Cl (pH 8.0) and sent for Illumina sequencing.

### MNase-seq data analysis and peak calling

The MNase-seq raw reads for wild-type *C. albicans* SC5314 cells grown under planktonic conditions were obtained from a previous study using the accession number GSE21960 (32). The MNase-seq data for wild-type *C. albicans* SC5314 cells grown under biofilm conditions were generated in-house. The quality of raw reads was first analyzed using FastQC (38). The required adapter sequences were trimmed using the tool cutadapt (39), and the quality check was performed again. The trimmed reads were aligned to the genome of *C. albicans* (assembly 22) using bowtie2 software (40). This was followed by .sam to .bam file conversion and sorting using SAMtools (41). PCR and sequencing duplicates were removed using Picard (42), and files were then visualized in Integrative Genomics Viewer (43). The .bam files for planktonic and biofilm data sets were normalized using deepTools software (44) using the RPGC method, which generated the .bigWig (.bw) files. The Bioconductor package, nucleR (33), was used for determining the nucleosome positions in planktonic and biofilm conditions. Briefly, .bam files after duplicate removal were imported into RStudio (45), and reads were centered according to the nucleosome dyads based on their coverage. The positions of nucleosomes were ultimately obtained after signal smoothing and peak calling as described in the package usage manual.

### Promoter analysis and plot generation

The TSS information for *C. albicans* genome was obtained from a previously published pipeline (20). Frequency distribution plot was generated for determining the distance between the TSS and the ORF start for *C. albicans* genome. The median promoter length (623 bp) of *C. albicans* genome was used from a previous publication (46). The computation for average profiles for nucleosome occupancy was performed using the computeMatrix method (44) using reference-point argument taking TSS or TFBS as the reference point. The average occupancy profiles were plotted using GraphPad Prism software (47).

### ATAC-seq data analysis

The raw reads obtained from Illumina sequencing were analyzed using FastQC (38). The adapter trimming was performed using fastx_trimmer from the FASTX-Toolkit (48). The trimmed reads were aligned to the genome of *C. albicans* (assembly 22) using bowtie2 software (40). This was followed by .sam to .bam file conversion and sorting using SAMtools (41). PCR and sequencing duplicates were removed using Picard (42). The nucleosome-free fragment (<100 bp) containing the .bam file was generated using a custom awk script. The .bam files for planktonic and biofilm data sets were normalized using deepTools software (44) using the RPGC method, which generated the .bigWig (.bw) files.

### Transcriptome data analysis

Raw values for microarray analysis were obtained from a previous publication (11). The statistical analysis was performed in GraphPad Prism software (47) on the raw values for the two replicates in planktonic and biofilm conditions. Multiple *t*-tests were performed with a false discovery rate (FDR) of 5% by using the Benjamin-Hochberg method. Only the genes showing a *q*-value <0.05 and log_2_(fold change) of >1.0 and <−1.0 were considered significantly upregulated or downregulated, respectively.

### ChIP-seq data analysis

The RNA polymerase II, H3K4me3, and H3K9ac ChIP-seq data sets were obtained from a previous publication (30). The H3V^CTG^ MNase ChIP-seq raw reads were generated in-house from cells grown in planktonic condition. The raw reads were trimmed using the fastp tool (49). The trimmed reads were aligned to assembly 22 of the *C. albicans* genome using bowtie2 software (40). This was followed by .sam to .bam file conversion and sorting the .bam file using SAMtools (41). PCR and sequencing duplicates were removed using Picard (42). The DNA fragment size for H3V^CTG^ MNase ChIP-seq data was restricted to mononucleosome length (150 bp) for downstream analysis. The log_2_ ratio of IP/Input was obtained using the bamCompare option from deepTools (44) software as .bigWig (.bw) output file. The 1× normalization was performed for H3V^CTG^ MNase ChIP-seq (input and IP files) using the bamCoverage option from deepTools software (44). H3V^CTG^-containing nucleosomes for eviction analysis were called using nucleR (33) as mentioned previously.

### Statistical analysis

The statistical significance of differences was calculated as mentioned in the figure legends. *P*-values ≥0.05 were considered nonsignificant. Precise *P*-values of the corresponding figures are mentioned in the figure panels or the figure legends, if significant. All statistical analyses were conducted using GraphPad Prism software (47).

## Data Availability

The raw reads obtained from sequencing experiments are submitted to SRA under the BioProject ID PRJNA980125.

## SUPPLEMENTAL MATERIAL

Fig. S1 mbio.02063-23-s0001.tif Biased genome-wide enrichment of H3VCTG is observed at promoters of *C. albicans* genes.

Fig. S2 mbio.02063-23-s0002.tif Promoters of genes whose expression remains unaltered in planktonic and biofilm cells are largely devoid of H3VCTG-nucleosomes in planktonic cells.

Fig. S3 mbio.02063-23-s0003.tif H3VCTG and histone H3 PTMs, which mark the transcriptionally active regions, bind in a non-overlapping manner at promoters of BR genes in planktonic cells.

Fig. S4 mbio.02063-23-s0004.tif Genome-wide nucleosome depletion corresponds to increased chromatin accessibility in biofilm cells.

Fig. S5 mbio.02063-23-s0005.tif Promoters of upregulated BR genes have increased open chromatin sites upon depletion of nucleosomes in biofilm cells.

Fig. S6 mbio.02063-23-s0006.tif Increased chromatin accessibility and depleted nucleosome occupancy is observed at promoters of downregulated BR genes in biofilm cells.

Table S1 mbio.02063-23-s0007.xlsx Genome-wide analysis of H3VCTG occupancy in planktonic cells.

Table S2 mbio.02063-23-s0008.xlsx List of differentially expressed genes during planktonic to biofilm transition.

Table S3 mbio.02063-23-s0009.xlsx Genomic coordinates of nucleosome occupied and open chromatin regions in planktonic and biofilm cells.

Table S4 mbio.02063-23-s0010.docx List of strains and primers used in this study.

ASM does not own the copyrights to Supplemental Material that may be linked to, or accessed through, an article. The authors have granted ASM a non-exclusive, world-wide license to publish the Supplemental Material files. Please contact the corresponding author directly for reuse.
