# Supplementary figures and images for: Biased eviction of variant histone H3 nucleosomes triggers biofilm growth in Candida albicans
Source: mBio. 2023 Sep 28;14(5):e02063-23. doi: 10.1128/mbio.02063-23 (PMC10653867; doi:10.1128/mbio.02063-23)

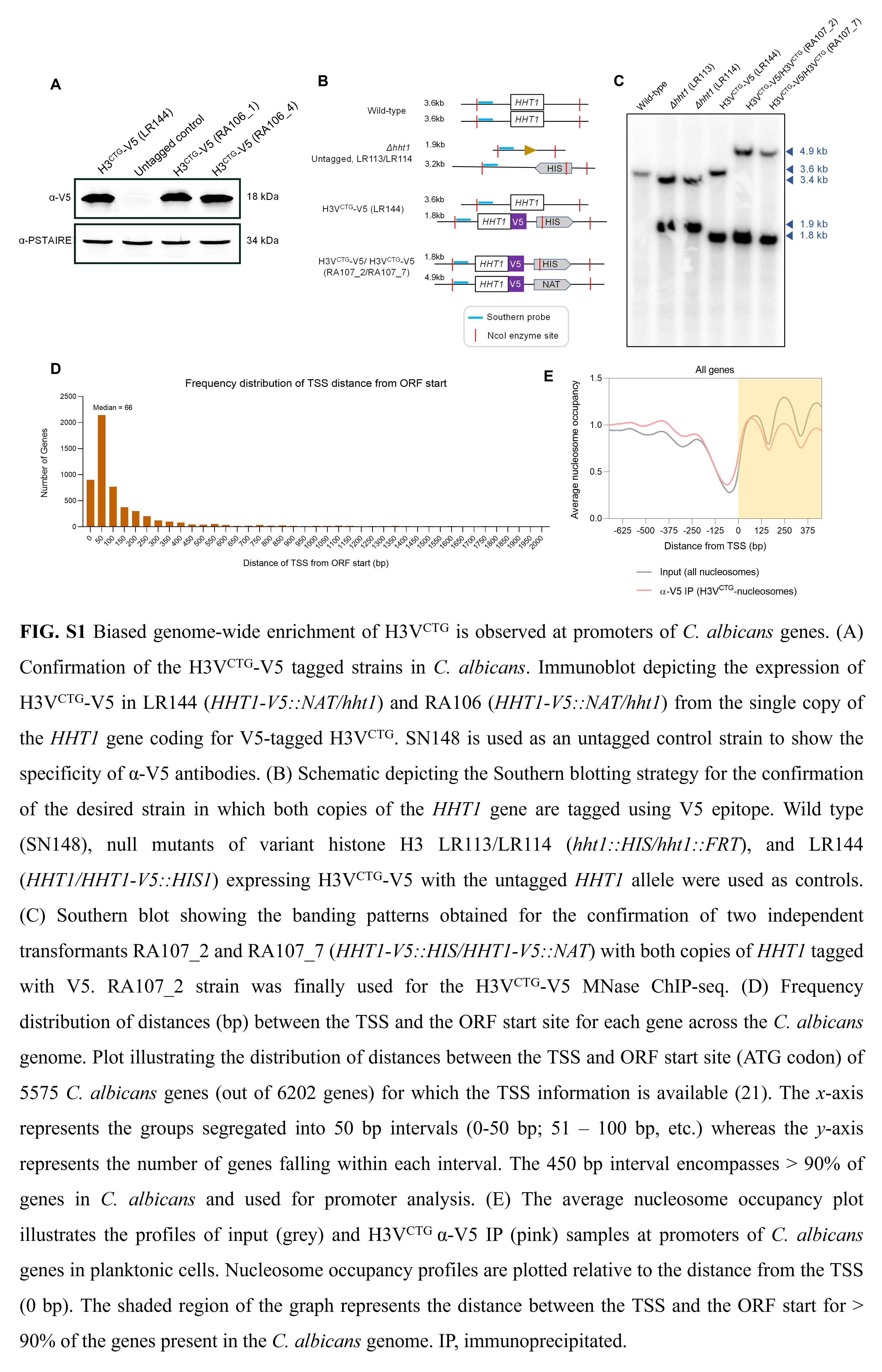

Supplement: Fig. S1 — Biased genome-wide enrichment of H3VCTG is observed at promoters of C. albicans genes. [file mbio.02063-23-s0001.tif]

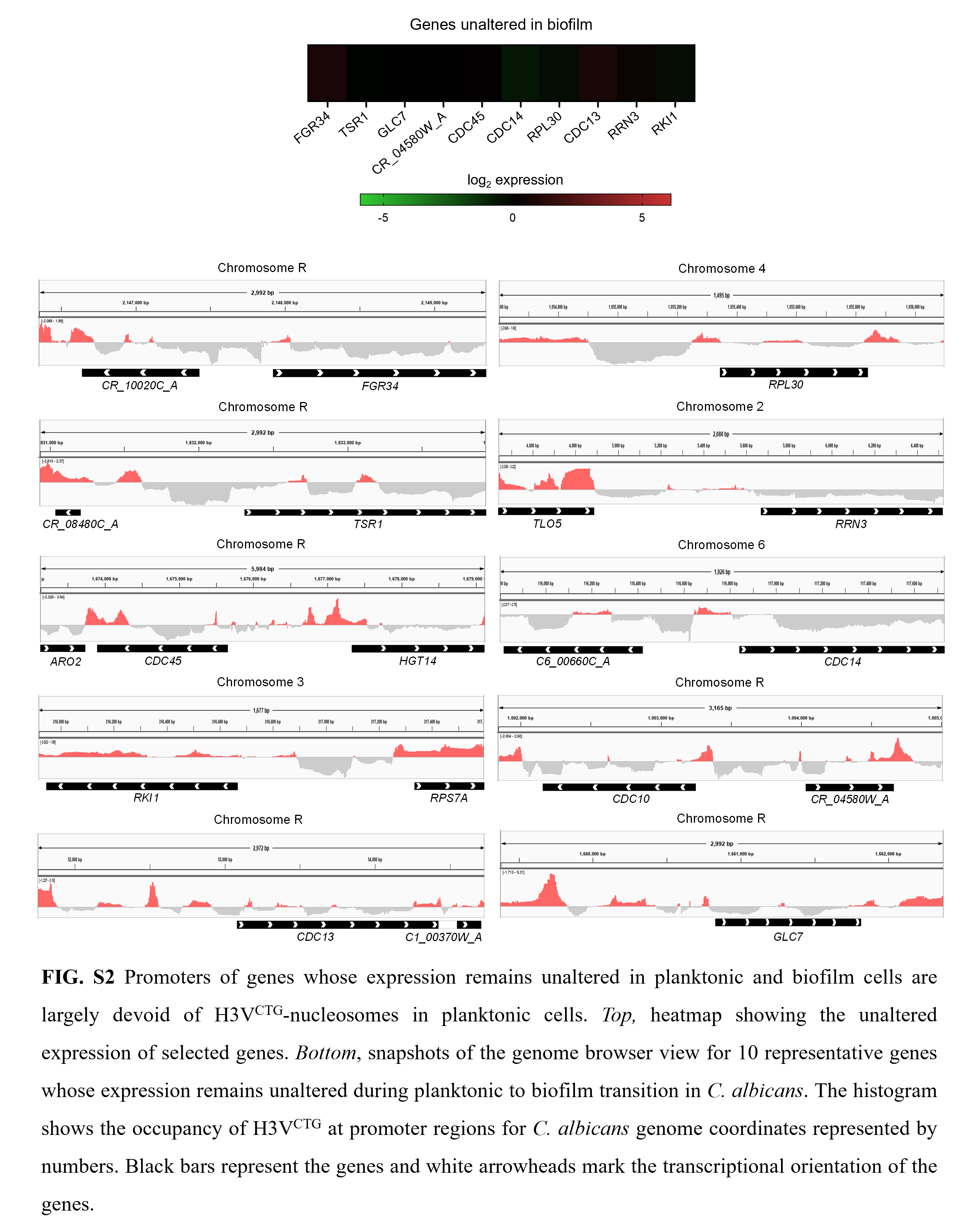

Supplement: Fig. S2 — Promoters of genes whose expression remains unaltered in planktonic and biofilm cells are largely devoid of H3VCTG-nucleosomes in planktonic cells. [file mbio.02063-23-s0002.tif]

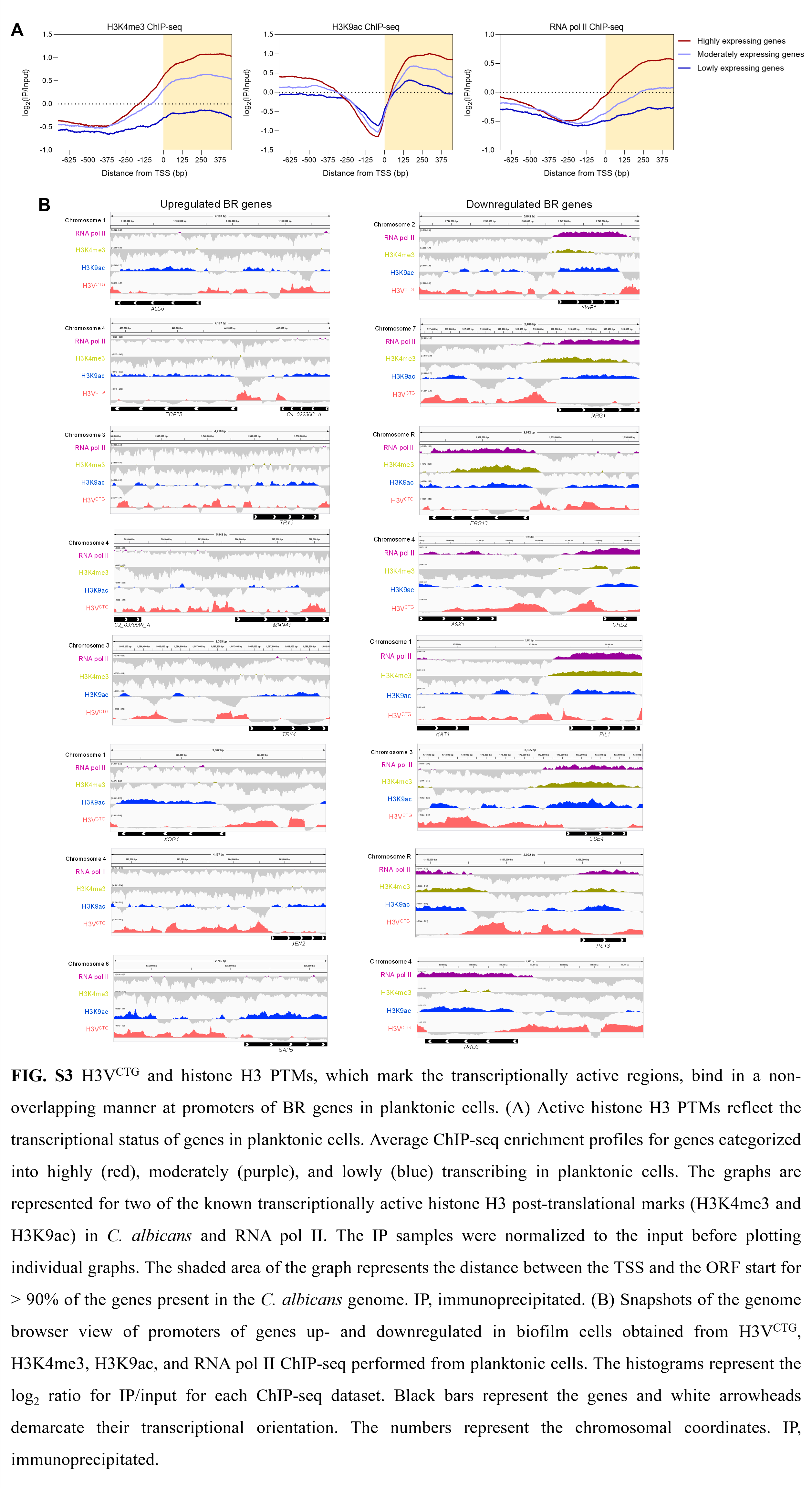

Supplement: Fig. S3 — H3VCTG and histone H3 PTMs, which mark the transcriptionally active regions, bind in a non-overlapping manner at promoters of BR genes in planktonic cells. [file mbio.02063-23-s0003.tif]

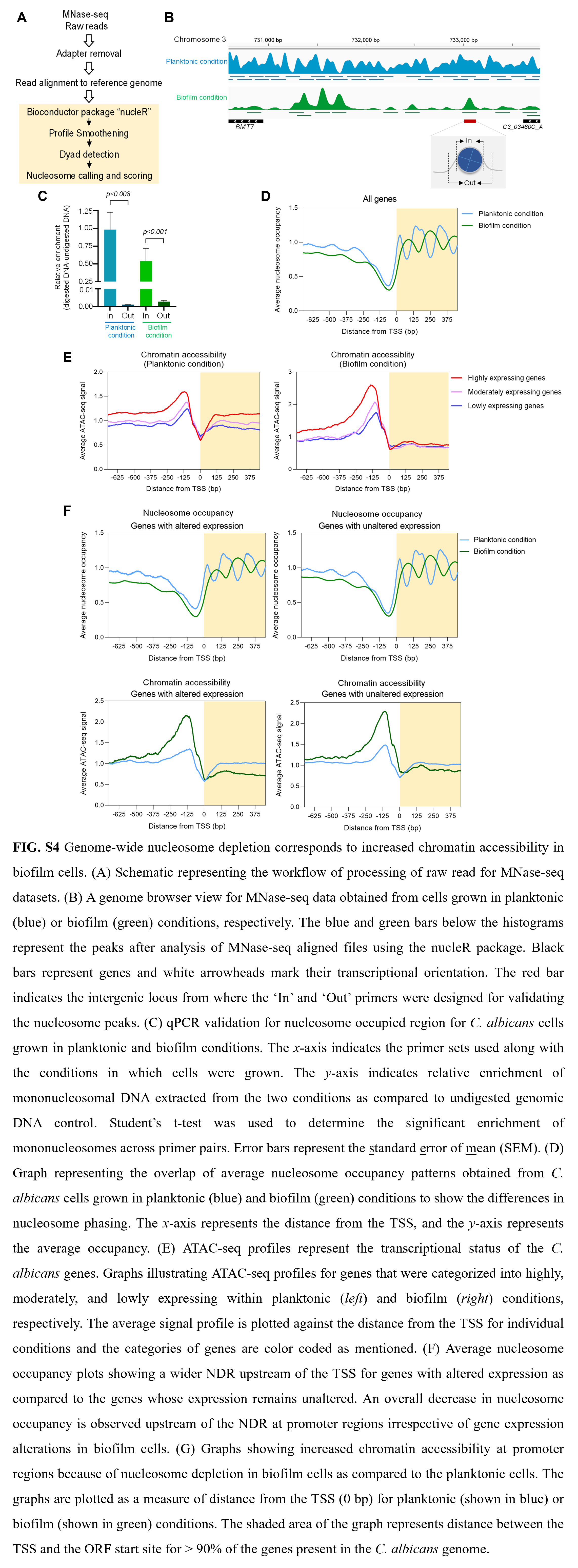

Supplement: Fig. S4 — Genome-wide nucleosome depletion corresponds to increased chromatin accessibility in biofilm cells. [file mbio.02063-23-s0004.tif]

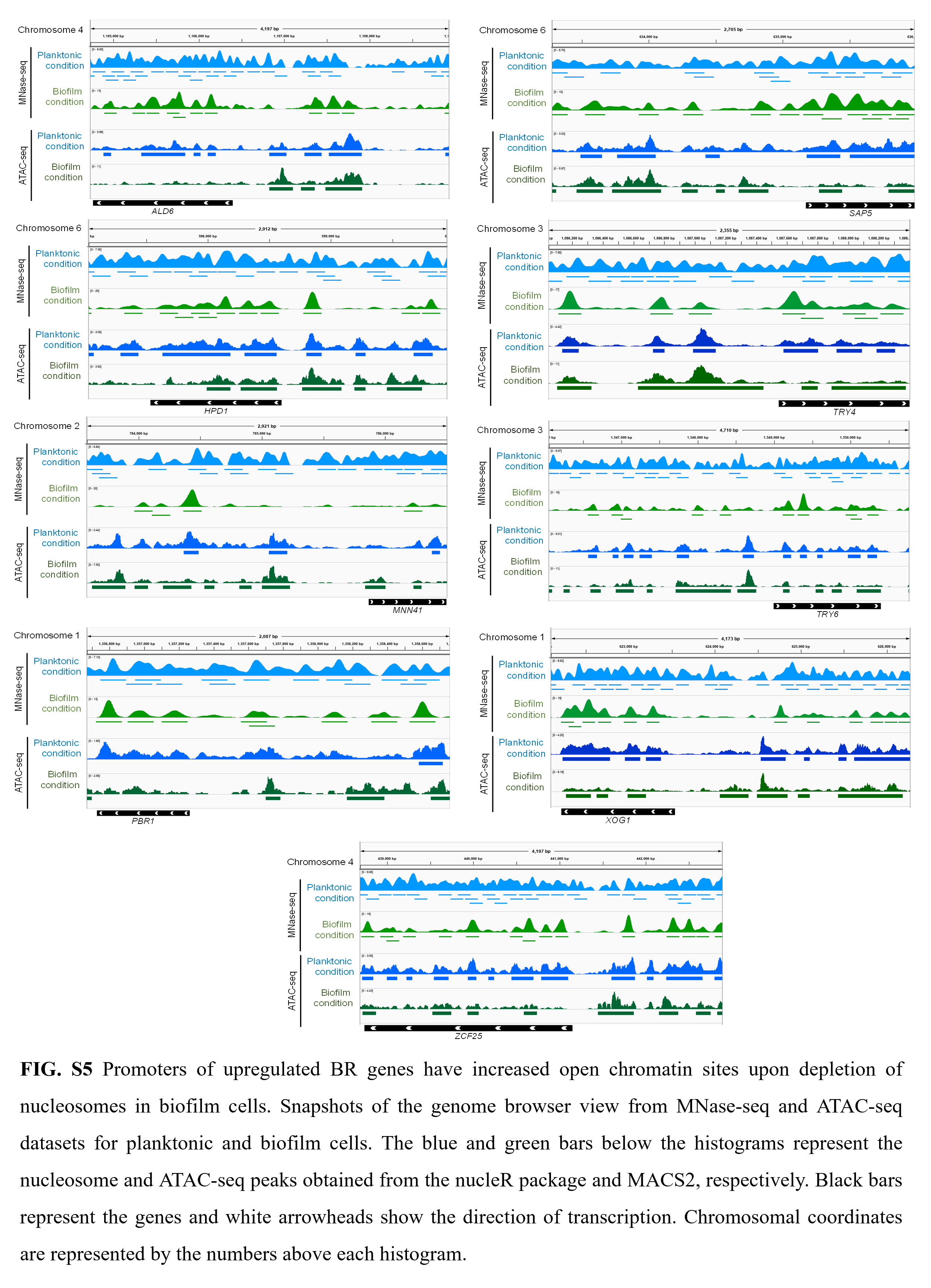

Supplement: Fig. S5 — Promoters of upregulated BR genes have increased open chromatin sites upon depletion of nucleosomes in biofilm cells. [file mbio.02063-23-s0005.tif]

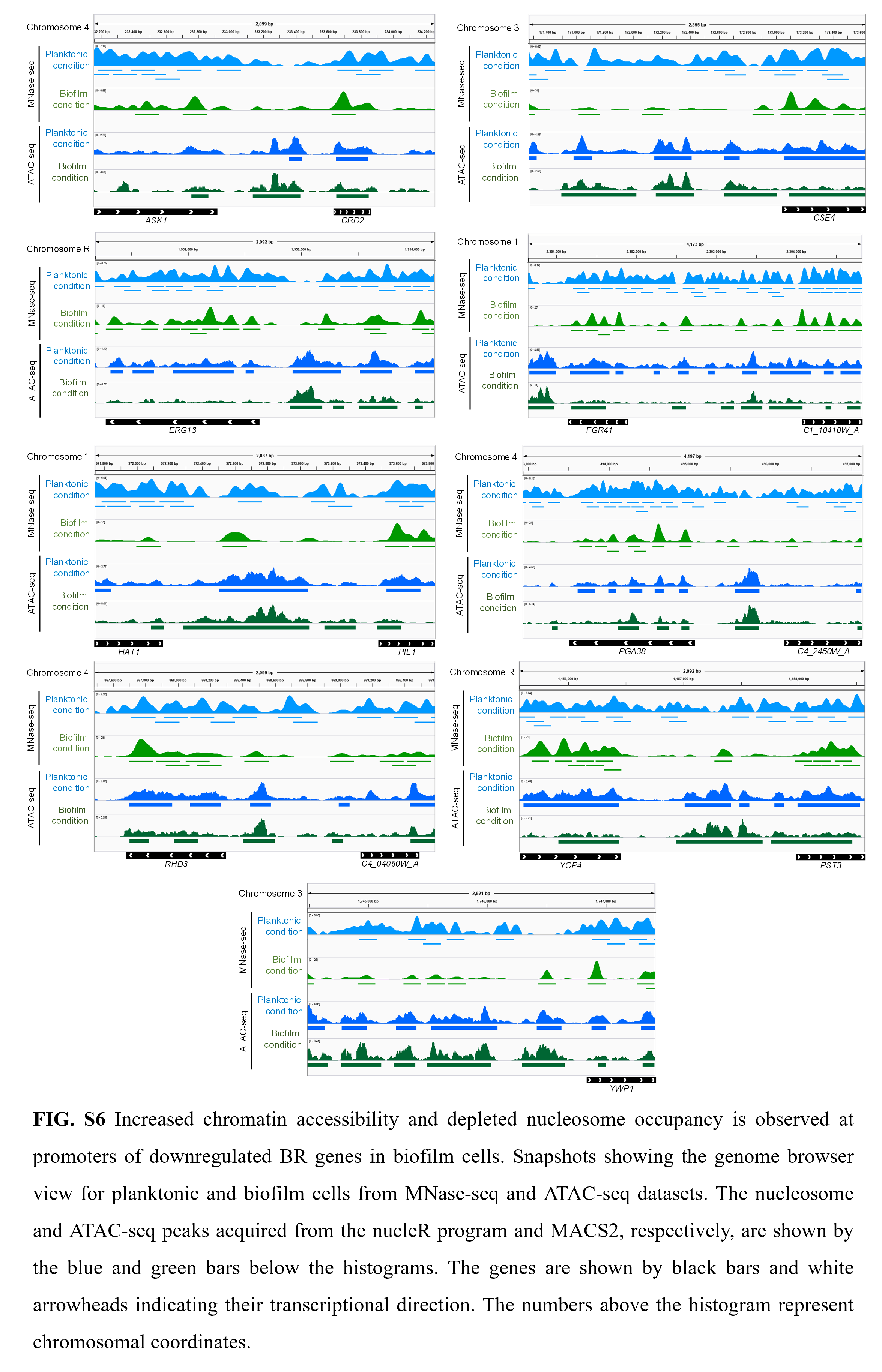

Supplement: Fig. S6 — Increased chromatin accessibility and depleted nucleosome occupancy is observed at promoters of downregulated BR genes in biofilm cells. [file mbio.02063-23-s0006.tif]
