# Supplementary material for: Biased eviction of variant histone H3 nucleosomes triggers biofilm growth in Candida albicans
Source: mBio. 2023 Sep 28;14(5):e02063-23. doi: 10.1128/mbio.02063-23 (PMC10653867; doi:10.1128/mbio.02063-23)
Supplement: Table S4 — List of strains and primers used in this study. [file mbio.02063-23-s0010.docx]

**Table S4A List of strains used in this study**

| Name (Description) | Genotype | Reference |
| --- | --- | --- |
| SC5314 | Wild-type clinical isolate | (1) |
| SN148 | *Δura3::imm434/Δura3::imm434, his1::hisG/Δhis1::hisG,*  *Δarg4::hisG/Δarg4::hisG,*  *Δleu2::hisG/Δleu2::hisG* | (2) |
| LR144 | SN148 *HHT1/HHT1-V5-HIS1* | (3) |
| LR113 | SN148 ∆hht1::HIS1/∆hht1::FRT RPS10/rps10::URA3 | (3) |
| LR114 | SN148 ∆hht1::HIS1/∆hht1::FRT RPS10/rps10::URA3 | (3) |
| RA106 | SC5314 *hht1::FRT/HHT1::V5-NAT* | This study |
| RA107 | SN148 *HHT1::V5-NAT/HHT1::V5-HIS1* | This study |

**Table S4B List of primers used in this study**

| Primer name | Sequence (5’-3’) |
| --- | --- |
| RS219 | GTGTAATACTGTAGCATTTGC |
| RS220 | CATTGCTATCCTTGACTATTAC |
| RS217 | GGCGGCAAAAAAAGTTTA |
| RS218 | CAATATGAACCAGAGTTTGC |
| Ad1_noMX | AATGATACGGCGACCACCGAGATCTACACTCGTCGGCAGCGTCAGATGTG |
| Ad2.1 | CAAGCAGAAGACGGCATACGAGATTCGCCTTAGTCTCGTGGGCTCGGAGATGT |
| 6791V5-CFP | CGAGCTCTTAAGAGGTGAAAGATCTGGTAAGCCTATCCCTAACCCTCTCCTCGGTCTCGATTCTACGTAAGACAGGATAAGATAGGAT |
| 6791V5-CRP | TCCCCGCGGGACTTCAAGATTATAATTAAAACAAAG |
| 6791V52LFP | CTAATTTATGTGCTATTCATGCTAAAAGAGTTACTATTCAAAAGAAAGATATGCAATTAGCTAGAAGATTAAGAGGTGAAAGATCT |
| RA1 | CACTCATTTGCAATTTCAGTAATTTATTATTCTACTTTTTAATATTTTTCCTTATGATTATCAACTCGGGCCCGGGACTGGATGGCGGCGTT |
| 6791SUSFP | CTCCAGCTCCATGGGCAAATAC |
| 6791USNAT1RP | CCGCTCGAGTATTGATTAAAAGTGTTGTTA |

REFERENCES

1. A. Aszalos, R. S. Robison, P. Lemanski, B. Berk, 55 g K2HPO4 I g. **XXI** (1968).

2. S. M. Noble, A. D. Johnson, Strains and strategies for large-scale gene deletion studies of the diploid human fungal pathogen *Candida albicans*. *Eukaryot. Cell* **4**, 298–309 (2005).

3. L. S. Rai, *et al.*, The *Candida albicans* biofilm gene circuit modulated at the chromatin level by a recent molecular histone innovation. *PLoS Biol.* (2019)
